# Physiology

**Published:** 1858-07

**Authors:** 


CHIEFLY FRENCH, GERMAN, AND ITALIAN; OR, BI-MONTHLY NOVELTIES IN MEDICAL, SURGICAL, OBSTETRICAL, AND CHEMICAL SCIENCE. BY EMIL FISCHER, M.D.

### PHYSIOLOGY.

### I. *Experimental Researches on the Physiological Properties and Uses of the Red and of the Black Blood.* By Dr. E. Brown-Séquard.

It is not the first time that M. Brown-Séquard has drawn the attention of physiologists to this subject, which his predecessors have but lightly touched, and in which so much still remains to be done. His researches commenced more than ten years ago, and he has published some of the principal results he had obtained in several communications made to the "Société de Biologie" and the "Académie des Sciences." In the treatise contained in the first number of his "Journal de la Physiologie de l'Homme et des Animaux," he compares the facts observed by him previously with those he has recently discovered, and proposes to give to the conclusions he believes himself justified in drawing from these different facts, the consideration which their importance deserve. The principal conclusions, drawn up in the form of propositions, of which each receives its experimental demonstration, are thirteen in number; the first five only are considered in the present article.

1. *The blood possesses two distinct physiological properties; the one of nutrition or of production of the vital properties of the tissues, the other of stimulation of the tissues and organs endowed with vital properties.*—The facts which demonstrate the existence of these two properties will be stated further below; here the author limits himself to define them, and to indicate the extent of their importance. Laying aside all considerations relative to secretions, he occupies himself only with the nervous and contractile tissues, or those having dynamic properties. Of the two physiological faculties which the blood possesses, there is one—that of maintaining the vital properties of these tissues, and even of regenerating them within a certain time after their complete disappearance—depending essentially on the oxygen it contains. Without oxygen the exchanges which take place between the tissues and the blood are not capable of maintaining the vital properties.

The other faculty is a stimulating or exciting property, such as that of heat, cold, or galvanism, when these physical agents, applied to contractile and nervous tissues, put them in action. The blood owes this important property to the carbonic acid it contains. By the first of these properties the blood thus gives life in power; by the other it gives life in action. With these general considerations M. Brown-Séquard connects the enumeration of the different details we shall meet as we proceed.

2. *The arterial and the venous blood differ essentially from each other, in their physiological properties, only on account of the quantity of oxygen and carbonic acid which these two fluids contain.*—The following experiment demonstrates that the nutritive property of venous blood differs from that of the arterial merely because it contains less oxygen.

*Experiment* 1.—120 grammes of arterial blood, and an equal quantity of venous blood, were defibrinized, and charged with oxygen by beating, and when both had become of equal scarlet-red color, they were injected, the arterial blood into the right femoral artery, the venous blood into the left femoral artery of a rabbit which had been dead for an hour, and in which the injected members had, ten or twelve minutes previously, attained cadaveric rigidity. The injections, made simultaneously, produced in both members the same effects at the same time; the rigidity ceased after five or six minutes, and restored muscular irritability manifested itself under the influence of shock, and of galvanism. Moreover, the irritability remained after the cessation of injection quite as long in the member which had received venous, as in that which had received arterial blood.

This experiment can be varied in different ways. When we deprive the arterial blood, after beating it, of a good part of its oxygen, through the agency of carbonic acid or hydrogen, this blood is incapable of regenerating muscular irritability, even soon after its disappearance; when we treat the venous blood, first oxygenized by beating, in the same manner, we arrive at the same result. Like results are obtained also, when, instead of acting upon the muscles, we try to recall the spontaneous actions of the nervous centres by means of these different kinds of blood.

The stimulating property depends alone on the quantity of carbonic acid contained in the blood. This is proved by the following experiment, in which these properties were, so to speak, inverted:—

*Experiment* 3.—In a rabbit which had been asphyxiated, and in which the irregular movements of the intestines, which are observed during and after asphyxia, were very strong, venous blood, highly charged with oxygen, was injected into the artery of one of the intestinal loops, the movements of which stopped very soon; in substituting immediately arterial blood charged with carbonic acid for this oxygenized blood, the movements were observed to return; another injection of oxygenized venous blood arrested them a second time, and they reappeared after another injection of black blood.

We shall find in another place of this memoir a great number of other facts which demonstrate the second proposition.

3. *All contractile tissues can, after having completely lost their vital properties, recover them under the influence of blood charged with oxygen, injected into the arteries.*—Thus the muscles of animal life can by this means be deprived of their rigidity, and again become irritable to the point of reproducing the phenomenon of muscular induction, and of recovering the power of engendering the galvanic currents, as practiced by Matteucci and Dubois-Raymond. The author has frequently proved the latter fact in batracchian and mammiferous animals. In regard, again, to the irritability after its complete cessation, and after the appearance of cadaveric rigidity, he has equally demonstrated the fact in two criminals. The following he observed in the second, a robust man, forty years of age, who was decapitated on the 12th of July, 1851, at 8 o'clock, A.M.:—

*Experiment* 5.—At 25 minutes past 10 o'clock in the evening all the muscles were rigid, and the last traces of irritability had disappeared We took about a pound of blood from the carotid of a vigorous dog; the blood was beaten and defibrinized, in order to avoid coagulation, and at 10 minutes past 11 o'clock the injection was commenced. It was made into the brachial artery of the left arm, at about its middle, where this extremity had been amputated. Soon after the commencement of the injection some reddish spots appeared on different parts of the skin of the forearm, and particularly on the wrist. These spots became larger and larger, and the skin appeared as it does in rubeola; soon afterwards the whole surface of the skin became violet-red. After some minutes this color disappeared, and gave place to the tint natural during life in a state of health. The skin became elastic and supple, as in the living individual, and we saw the hair-bulbs erecting themselves, and the cutis anserina produced. By augmenting and diminishing alternately the impulse of the current of blood, we succeeded in producing movements similar to the pulse in the radial artery; the subcutaneous veins were distinctly visible, and seemed as full as during life. Sometime after the commencement of the injection (twelve or fifteen minutes) the fingers, which had been very stiff, were relaxed, and the rigidity disappeared also in the other parts of the member. At a quarter to 12 o'clock (after thirty-five minutes of injecting) the irritability was restored, particularly in the muscles of the arm. It existed still in all the muscles of the member at 4 o'clock, A.M., the next day, (twenty hours after decapitation.)

In the first criminal nearly the same circumstances took place. In animals the results are similar, and can be varied much more by the experiments.

In comparing these results with those of the experiments made on man, M. Brown-Séquard proved that irritability disappeared later, and that cadaveric rigidity came on later in man than in animals. The maximum duration of muscular irritability in rabbits has been eight hours and a half, and in Guinea-pigs eight hours and some minutes; in dogs it is sometimes nine or ten hours. In one of the two criminals referred to the irritability had completely disappeared in ten or twelve hours, and in the other in about fourteen hours after death.

The results, however, of a number of experiments, show that the more energetic the irritability is at the moment of death, the easier and the longer possible it is to recall it after the establishment of cadaveric rigidity.

In the same manner as with the other muscles of animal life, can the heart become irritable again, after having completely ceased to be so; but of all the contractile organs it seems to be the least capable of reacquiring irritability. What is true in regard to the striated muscles, is equally so in reference to those of organic life; the intestine, stomach, œsophagus, uterus, iris, bladder, ureters, seminal vesicles, blood-vessels, and thoracic duct, the contractile fibres surrounding the hair-bulbs in man, can recover their irritability after having lost it.

4. *All the nervous tissues can, after having completely lost their vital properties, recover them under the influence of blood charged with oxygen.*—The correctness of this proposition is demonstrated, in regard to the motor and sensitive nerves, by the following experiment:—

*Experiment* 9.—The abdomen of a dog was opened by a nearly complete circular section, and the aorta ligated above the origin of the renal arteries; then a linen band was stitched to the lips of the abdominal wound, so as to avoid exposure of the viscera to air. After the lapse of nineteen minutes, sensibility had completely disappeared in all parts of the skin of the posterior extremities; after twenty-four minutes voluntary motion was abolished. About half an hour afterwards the sciatic nerves, when directly excited by galvanism, did not give any sign of excitability; the irritability of the superficial muscles had disappeared in an hour and thirty-six minutes after the operation. Cadaveric rigidity commenced to show itself a little afterwards, and in two hours after the application of the ligature it was very strong and general. The ligature was then cut, and circulation re-established. The rigidity ceased, and in six or eight minutes muscular irritability reappeared. The motor excitability of the sciatic nerves commenced to manifest itself in about twenty minutes after the re-establishment of the circulation. In twenty-eight minutes after the section of the ligature sensibility was restored, and there were, at the same time, feeble appearances of voluntary or reflex movements. An hour later the posterior members made quite energetic voluntary movements, and the sensibility had been restored in them in an almost normal degree.

The spinal marrow in the same way can recover its vital properties; this is proved by an experiment similar to the preceding, by dividing the spinal marrow at a level with the second or third lumbar vertebra in such a way as that the inferior extremity of this organ, separated from the encephalon, does not receive any more blood after the ligation of the aorta. It is found, then, that the reflex faculty is quickly lost, and that the excitability of the lumbar enlargement disappears some time before the motor nerves lose their vital property. After the restoration of the circulation the excitability of the lumbar region of the spinal marrow, and its faculty to produce reflex motions, reappears some time after the return of the properties of the motor nerves.

5. *The brain, after having completely lost its functions and vital properties, can recover them under the influence of blood charged with oxygen.*—M. Brown-Séquard supports this proposition by experiments, in which he tied the two carotids and vertebral arteries in animals. In two or three minutes after cutting the ligature, he saw all the cerebral functions restored; but, says he, experiments like the following one demonstrate still better this influence of the blood on the brain and medulla oblongata:—

*Experiment* 11.—I decapitated a dog, taking care to make the section below the point where the vertebral arteries enter their osseous canals. Eight minutes afterwards, pinching of the skin having no effect, I applied a galvanic current of considerable intensity to the medulla oblongata, previously exposed, taking care to avoid passing the current through neighboring parts. No movement became manifest. The conductors, applied to the protuberance, produced no effect. Ten minutes after the cessation of the respiratory movements of the nostrils, lips, and inferior maxilla, I adapted to the four arterial trunks of the head, canulas, which were connected by tubes of caoutchouc, with a copper cylinder, through which I injected, by means of a syringe, blood charged with oxygen. In two or three minutes, after some slight irregular movements, I saw movements of the eyes and muscles of the face, which seemed directed by the will.

I prolonged the experiment for a quarter of an hour, and during all this time these movements, apparently voluntary, continued. The injection being stopped, these movements ceased, and were soon replaced by convulsions of the eyes and face, by respiratory movements of the nostrils, lips, and maxilla, and finally, by the tremors of death. The pupil was contracted and then dilated, as in ordinary death.

M. Brown-Séquard endeavors then to prove that the regeneration of vital properties has not been shown by any experiments anterior to his. In the apparent death of new-born and asphyxiated animals, these properties are not lost; they have, therefore, not been regenerated by calling back life by pulmonary insufflation or other means.

Stenon, Bichat, and other physiologists, have proved the abolition of sensibility and voluntary motion after the ligature of the aorta, and their return after the re-establishment of the circulation; but they have not studied the influence of the circulation upon the different tissues, muscles, and nerves.

Kay announces, it is true, that he has seen irritability returning in muscles which had ceased to contract under the influence of galvanism, by injecting blood into their arteries. "But," says M. Brown-Séquard, "this distinguished physiologist did not carry his experiment far enough; he believed muscular irritability lost, because he did not see motion. This was not a sufficient sign; muscles, if stimulated, can preserve irritability half an hour, or even longer, after movements have ceased to be produced; contractions take place in a great part of a galvanized muscle without the movement of the member in which this muscle is situated. To be certain that muscular irritability has completely disappeared, it must be shown not only that galvanism and shocks do not produce any more contraction, but also, that cadaveric rigidity is established. Besides, if the irritability had disappeared in reality in the experiments of Kay, he did not succeed in restoring it by injections of venous blood, as he believed he had; for the venous blood is absolutely incapable of regenerating the vital properties of muscles by the time they have completely disappeared. This will be proved in the continuation of this memoir."—(*Archives Générales*, March, 1858; from *Journ. de Physiologie*, 1858, No. 1.)

II. *Memoir on the Effects of Extirpation of the Pancreas.* By PROF. BÉRARD and M. COLIN. (Read before the Académie de Médecine, Jan. 19th, 1858.)

The experiments, of which this memoir gives an account, were made by MM. Colin and Bérard, at the school of Alfort, and the results which they furnished were verified repeatedly by a commission composed of MM. Duméril, Cloquet, Jobert, Wurtz, Longet, Ségalas, and Renault. The object of the experimenters was to ascertain whether extirpation of the pancreas, or ligation of its excretory ducts, produced the same effects in carnivorous as in herbivorous animals.

The first experiments were made on dogs; after some preparatory trials, the following mode of proceeding was adopted as the best:—The animal was left without food for four or five days; then an opening was made through the abdominal parieties, in order to draw out the loop of the duodenum, to which the pancreas is fixed. The pancreatic ducts were ligated, and oil and warm water were injected successively into the cavity of the intestine, through a puncture made for the purpose. The duodenum and pancreas were pushed back into the abdominal cavity, and the external wound closed by sutures. After three or four hours the animal was killed. Sixteen dogs were thus sacrificed, and in all white, opaque chyle was found in the mesentery, and even in the walls of the intestines. But the result of these experiments might be considered inconclusive, on the ground that the pancreatic juice found in the intestine of these animals might have been retained there from a previous digestion. In order to remove this objection, two palmipeds, two pigs, and several dogs were operated upon in May and June, 1857, and not killed until several months after the operation.

In the duck the duodenum forms an elongated loop, and the two lobes of the pancreas, which unite at their superior part, fill up the space. On opening the duck which had served for the experiment, it was found that of this gland nothing was left but two small tubercles, which had not the least connection with the intestine; the animal had lived eight months after having lost its pancreas; at the time of the operation it weighed 730 grammes; six months later its weight had increased to 1667 grammes, and its body contained a fair proportion of fat.

A goose, which had been operated upon in the same way, died at the end of six months; it is probable that it might have been saved by substituting, as in the duck, a strongly nitrogenized regimen for an exclusively vegetable food.

In the two pigs all the internal part of the pancreas was extirpated; it was, therefore, certain that the duodenum did not receive any more pancreatic juice, and the fact was proved by the autopsy; the animals had, nevertheless, increased in size. The second had gained twenty-five kilogrammes in five months and a half; and on opening its body the mesenteric vessels were found to contain emulsified chyle, and the layer of fat deposited on its back measured three centimetres in thickness.

Finally, five very young dogs, of the same litter, were operated upon about eight months ago. Two are yet alive, and are fine-looking, perfectly developed animals. In the others, which were not inferior in development, the autopsy proved the pancreas to be totally isolated from the intestine, and its canal obliterated. There remained only a small granulation, of the size of a bean, which opened separately into the intestine, and which had no communication at all with the gland proper. Could this organ have supplied the place of the pancreas? In order to show the inadmissibility of this supposition, it is sufficient to refer to the weight of the structure, which was about the ninetieth part of that of the pancreas in a dog of the same size.

"Our vivisections," says M. Bérard, in conclusion, "have proved to us that fatty matters can be digested and absorbed without the participation of the pancreas; that under these conditions the animals live very well, are regularly developed, grow fat, have no fatty evacuations, and preserve the mucous membrane of their digestive organs in good condition, and with large and perfect villosities.

"All these results of experiments, which have been followed up for a long time, and multiplied on various types of animals, permit us now to generalize more than formerly, and to say, without rashness, that *in herbivorous, ruminant animals, in carnivorous, in the pig, which is omnivorous, and in birds, the pancreatic fluid is not necessary either for the digestion or the absorption of fatty substances.*"—(*Gaz. Hebdomad.*, Jan. 22d, 1858, and *Gaz. Méd de Paris*, Jan. 1858, No. 4.)

III. *On the Nervous Centres controlling the Contraction of the Uterus.* By DR. O. SPIEGELBERG, of Göttingen.

As in the physiology of the nervous system of all the other viscera, so in that of the uterus, the question of the locality of its nervous centres remains still open to dispute. Some physiologists (Brachet, Longet, Valentin, Budge, Franz M. Kilian) referred these centres to one or the other part of the cerebro-spinal system, others (Berling, H. Nasse) to the sympathetic, and vindicated their opinion by experimental researches.

The author experimented on forty animals, mostly rabbits, some cats, and a few guinea-pigs. He communicates the results obtained in thirty-five cases.

In experimenting upon organs endowed with organic muscular fibres, it is very important for the physiologist to be able to exclude the spontaneous motion in these organs from that produced by the irritation of certain parts of the nervous system. Some preliminary experiments, made partly for this purpose by the author, confirmed the fact that cessation of circulation produced always peristaltic contraction of the uterus, while this organ remained at rest as long as the heart pulsated, no matter whether air was admitted into the parts or not. Dr. Spiegelberg believed he had thus found a pretty reliable criterion for the discrimination between spontaneous contractions and those resulting from irritation, and therefore made his experiments and observations on living animals. On the same ground he considers the experiments of Kilian as doubtful in their results, as they were made on dead animals, in which even the preservation of the peritoneum is insufficient to prevent peristaltic action.

The animals were put under the influence of ether before the operation. In laying bare the parts, loss of blood was carefully avoided. For the purpose of irritating the parts of the nervous system exposed, Dubois' induction-apparatus was used. The cerebellum, and frequently also the spinal cord, had to be excited by mechanical and chemical irritants, as in their case isolation of the electric influence is impossible.

The author sums up the results of his experiments in the following propositions:—1. *Cessation of circulation and the stasis of blood produced by it are the causes of the peristaltic movements of the uterus. As long as the heart pulsates, these movements are not observed at all, or are but exceedingly slight.* The deportment of the uterus resembles in this respect that of the intestinal tube. It is possible that anæmia and hyperæmia play a part in this phenomenon. We are inclined to lay more weight upon anæmia from the following causes:—(*a.*) In animals which die from loss of blood, the abdominal viscera in general, as well as the uterus, show more vigorous peristaltic motions. (*b.*) After the death of the animal a stronger peristaltic action is always observed in that cornu uteri the vessels of which have been emptied by separation from the mesometrium. (*c.*) In repeated experiments on living animals compression of the aorta immediately below the diaphragm was always followed by contractions of the genital organs and the intestines, which lasted as long as the blood going to these organs was arrested; when the impediment was removed, rest was gradually restored in them.

2. *Through the par vagum no impressions are conveyed to the uterus,* although

Franz M. Kilian established the contrary as the result of his experiments. If, after irritating the pneumogastric, motions of the uterus were observed, they were of a spontaneous kind, or owing to the paralyzing influence exercised upon the heart; for, if the irritation was so feeble as not to affect the heart, contractions of the uterus were not produced. When irritation of the par vagum is followed by uterine contractions, it acts in the same manner as the compression of the abdominal aorta. The failure of this experiment was particularly conspicuous in animals which show a feeble power of peristaltic action in general, as in cats and dogs, while, however, upon irritation of the nervous centres a vigorous reaction in the genital organs was produced. With this view harmonize also the results of irritation of the nerve after its ligation; irritation made at its peripheral end produced no effect, except when the heart was affected; that at the central end, however, influenced the uterus, undoubtedly through the agency of the medulla oblongata; in fact almost each time the abdominal muscles were simultaneously thrown into contraction, a phenomenon never observed in consequence of irritation of the peripheral end.

3. *Uterine contractions can be produced by irritation of the medulla oblongata.*—As this irritation has a similar effect upon the heart as that of the par vagum, it remained to be proved that the excitation of the uterus is not a secondary phenomenon; but although the pneumogastric was compressed and cut through, irritation of the medulla oblongata produced the same effect upon the uterus. The result was also equal no matter whether the heart was affected or not.

4. *The principal centre is the cerebellum.* Irritation of its central as well as of its lateral parts, be it superficial or deep, was always followed by uterine contraction. Sometimes it was also observed after irritation of the posterior corpora quadrigemina, but not after that of the cerebrum. The author confirms thus the opinion of Budge and Valentin that the motor nerves of the uterus have their central ends in the cerebellum.

5. *From each portion of the spinal cord, but more especially from the lumbar and sacral parts of the same, contractions of the uterus can be excited.* In unimpregnated animals, irritation of the spinal cord excited uterine contractions, or increased them if they already existed. In pregnant animals the existing contractions of the uterus ceased if the spinal cord was irritated. In regard to the latter phenomenon, the author adopts Schiff's explanation of the arrest of motion observed in consequence of the irritation of nerves; this physiologist assumes that this phenomenon is not a result of activity of the nerve, but is, on the contrary, owing to excessive excitation or exhaustion. This view being taken, the results of irritation of the spinal cord in pregnant animals serve to prove the foregoing proposition just as much as those obtained in unimpregnated animals.

6. *Irritations produced in the central parts are conducted along the medulla oblongata and the spinal cord downward, and reach the uterus through the branches of the sympathetic nerve connected with the cord and through the sacral nerves; while irritations originating in the uterus travel the same route in an opposite direction to the central parts.*—This proposition is rather inferred from foregoing reasons than proved by experiments. As irritations originating in the cerebellum are not conveyed along the par vagum, but go downward along the spinal marrow, which has only sympathetic connections with the abdominal viscera, the bridge from the spinal marrow to the uterus has to be sought for in the communicating branches. Irritation of the sympathetic in the thoracic cavity was never followed by marked effect; irritation of the sympathetic and its connecting branches in the abdominal cavity was more successful; but the author lays no stress upon this circumstance, as the excitation in this case cannot be directed wholly upon the nerve. The obvious influence of the lumbar and sacral parts of the spinal marrow as well as of the trunk of the sympathetic, which is confirmed by all observers, he considers of great importance.

In case the above proposition is correct, the increase and diminution of the frequency of the pulse, observed by Martin and Maurer in connection with labor-pains, is not to be explained by the excitation of the uterus being transmitted through the sympathetic and par vagum to the heart; but by its being conducted along the cord to the medulla oblongata, whence it is transferred to the pneumogastric, thus producing an increase of the heart's action.

Finally, the author observes that he by no means attributes the incitement of parturition to the nervous system. The influence which the disturbance of the circulation has in producing uterine contractions, as shown by his experiments, leads him, on the contrary, to the hypothesis that local changes in the circulation of the uterus, taking place at the termination of gestation, are the causes of the beginning of contraction.

In regard to the mode of uterine contractions, which, as they are of the peristaltic kind, cannot exist in the whole organ at once, the author has observed the following facts:—At first the mesometrium contracts and fixes the uterus against the pelvis; then the vagina and the cervix uteri become narrower, and this circular contraction is propagated to one or both cornua uteri, until a strong constriction is formed over the fœtus lying next to the cervix; this during a simultaneous dilatation of the vagina and cervix progresses downward, and propels the fœtus in the same direction. The true expulsive contraction therefore commences in the upper part of the uterus; previous to it, however, the organ is fixed to the pelvis by the broad ligaments, and by its own longitudinal fibres.

In woman the uterus is probably fixed during each pain by the round ligaments, and by the muscular fibres of the broad ligaments against the pelvis, (hence its so-called erection, and its greater protrusion from the anterior abdominal parietes;) and then the fundus contracts over the ovum and propels it downward:—(*Schmidt's Jahrb.*, 1858, No. 3, from *Henle und Pfeufer's Ztschr.;* 3 Reihe, ii., p. 1–43, 1857.)

IV. *On the Peristaltic Motion of the Intestines.* By DR. SPIEGELBERG.

The experiments made with reference to the contraction of the uterus, served the author with observations in regard to the peristaltic motions of the intestines. His attention was directed principally to three points:—

1. *The dependence of intestinal contractions upon the spinal cord and brain*, a known fact, was fully confirmed. By mechanical as well as electric irritation of each point from which the uterus was influenced, the intestine also could be put into motion.

2. *The influence of disturbed circulation upon the intestines, especially the small, is exhibited in a still higher degree than in the uterus.*—As soon as the circulation commences to stagnate, vigorous peristaltic motions take place, and increase with its complete cessation. Compression or ligation of the abdominal aorta below the diaphragm, acts in the same manner as arrest of circulation. After ligation of the vena cava or vena portarum, peristaltic motions never became as vigorous as after ligation of the aorta; a circumstance which speaks for the greater importance of anæmia in producing the phenomenon. Each change of circulation, in general, is capable of producing peristaltic motions of the intestines. These facts are contradictory to the opinion of Brown-Séquard, who believes that the carbonic acid accumulated in venous blood is the exciting agent. To ascribe the peristaltic motions observed, after opening the abdominal cavity in recently killed animals, to the irritation produced by the access of atmospheric air, is no doubt a great error.

3. *Pflüger's new method of checking peristaltic motions of the intestines by irritation of the spinal marrow was practiced repeatedly with success.*—(*Henle und Pfeufer's Ztschr.* 1857; 3 Reihe, ii., p. 44.)

---

